# Supplementary material for: The B.1.427/1.429 (epsilon) SARS-CoV-2 variants are more virulent than ancestral B.1 (614G) in Syrian hamsters
Source: PLoS Pathog. 2022 Feb 10;18(2):e1009914. doi: 10.1371/journal.ppat.1009914 (PMC8865701; doi:10.1371/journal.ppat.1009914)
Supplement: S2 Table — (DOCX) [file ppat.1009914.s002.docx]

| S2 Table. Primer sequence for QUILLS assays used in this study | | |
| --- | --- | --- |
| Primer designation | sequence | Tm |
| spike-K417-L452-F | ACAAATCGCTCCAGGGCAAACTGG | 59.1 |
| spike-K417-L452-R | CTCTCTCAAAAGGTTTGAGATTAGACTTCC | 58.9 |
| spike-E484-N501-A570-D614-F | TATCAGGCCGGTAGCACACCTTGT | 59.1 |
| spike-E484-N501-A570-D614-R | GCAACAGGGACTTCTGTGCAGTTAAC | 59.5 |
| spike-H655-P681-F | ACACGTGCAGGCTGTTTAATAGGGG | 59.3 |
| spike-H655-P681-R | ATTGACTAGCTACACTACGTGCCCG | 59.3 |
| orf1ab-P976-D1183-F | TCTTGTCTGTTAATCCGTATGTTTGCAATGC | 59.1 |
| orf1ab-P976L-D1183-R | CAGGTGGTCCCTGGAGTGTAGAATA | 59.3 |
| spike-T1027-V1176-F | ATTAATTAGAGCTGCAGAAATCAGAGCTTCTG | 59.3 |
| spike-T1027-V1176-R | TTCTTGGCAACCTCATTGAGGCGGT | 59.3 |
