# Supplementary material for: The B.1.427/1.429 (epsilon) SARS-CoV-2 variants are more virulent than ancestral B.1 (614G) in Syrian hamsters
Source: PLoS Pathog. 2022 Feb 10;18(2):e1009914. doi: 10.1371/journal.ppat.1009914 (PMC8865701; doi:10.1371/journal.ppat.1009914)
Supplement: S1 Source Data — Fig 1A Data. Source data used in Fig 1A. Fig 1B Data. Source data used in Fig 1B. Fig 1C Data. Source data used in Fig 1C. Fig 5A Data. Source data used in Fig 5A. Fig 5B Data. Source data used in Fig 5B. Fig 5C Data. Source data used in Fig 5C. Fig 5D Data. Source data used in Fig 5D. Fig 6A Data. Source data used in Fig 6A. Fig 6B Data. Source data used in Fig 6B. Fig 7A Data. Source data used in Fig 7A. Fig 7B Data. Source data used in Fig 7B. Fig 7C Data. Source data used in Fig 7C. Fig 7D Data. Source data used in Fig 7D. Fig 7E Data. Source data used in Fig 7E. Fig 8A Data. Source data used in Fig 8A. Fig 8B Data. Source data used in Fig 8B. Fig 8C Data. Source data used in Fig 8C. Fig 8D Data. Source data used in Fig 8D. Fig 9AB Data. Source data used in Fig 9. S1A Fig Data. Source data used in S1A Fig. S1B Fig Data. Source data used in S1B Fig. S1C Fig Data. Source data used in S1C Fig. (ZIP) [file ppat.1009914.s005.zip › S1 Source Data/Fig6A-table.pdf]

Table format: Grouped		Group A							
		614G							
		A:Y1	A:Y2	A:Y3	A:Y4	A:Y5	A:Y6	A:Y7	A:Y8
1	Day 2	0.0	0.0	0.0					
2	Day 4	0.0	0.0	0.0					

	Group B									
	427									
	B:Y1	B:Y2	B:Y3	B:Y4	B:Y5	B:Y6	B:Y7	B:Y8	C:Y1	C:Y2
1	1.0	1.0	1.0						*0.29**	*0.37**
2	1.0	1.0	1.0						0.53	0.21

	Group C								
	614G/427 1:1								
	C:Y3	C:Y4	C:Y5	C:Y6	C:Y7	C:Y8	D:Y1	D:Y2	D:Y3
1							0.30	0.15	0.21
2	0.42						0.08	0.08	0.35

	Group D				
	614G/427 9:1				
	D:Y4	D:Y5	D:Y6	D:Y7	D:Y8
1					
2					
