# Supplementary material for: The B.1.427/1.429 (epsilon) SARS-CoV-2 variants are more virulent than ancestral B.1 (614G) in Syrian hamsters
Source: PLoS Pathog. 2022 Feb 10;18(2):e1009914. doi: 10.1371/journal.ppat.1009914 (PMC8865701; doi:10.1371/journal.ppat.1009914)
Supplement: Supplementary file 25 [file ppat.1009914.s001.docx]

Supplemental Table 1. Histology scoring criteria

**Airway % affected (0-4)**	(0) none<br>(1) <10%<br>(2) 10-25%<br>(3) 25-50%<br>(4) >50%
**Airway severity (0-4)**	(0) Within normal limits (or rare/minimal peribronchial/peribronchiolar mononuclear infiltrates not observed in control animals but significance questionable)<br>(1) Mild peribronchitis/bronchiolitis<br>(2) Mild to moderate mononuclear to mixed peribronchiolitis and/or lumens contain low numbers of inflammatory cells and/or multifocal single cell necrosis of airway epithelium<br>(3) Moderate to marked mixed peribronchitis or bronchiolitis, and/or large foci of bronchiolar epithelial necrosis, and or bronchiolar epithelium exhibit occasional atypical/multinucleated syncytial cells<br>(4) Marked bronchiolitis and widespread epithelial necrosis +/- rupture of bronchiolar epithelium and/or bronchiolar epithelium exhibits frequent atypical/multinucleated syncytial cells
**Bronchial epitheial hyperplasia (0-3)**	(0) None<br>(1) Sporadic bronchiolar epithelial hyperplasia (characterized by piling up and increased mitoses) affecting less than 10% of the section's airways<br>(2) Mild to mod BEC hyperplasia affecting 10-25% of the section's airways<br>(3) Widespread BEC hyperplasia and/or atypical/multinucleated syncytial cells affecting greater than 25% of the section's airways
**Total airway score**	**0-11**
**Alveoli % affected (0-4)**	(0) None<br>(1) <10%<br>(2) 10-25%<br>(3) 25-50%<br>(4) >50%
**Alveolar severity (0-4)**	(0) Within normal limits (or rare/minimal peribronchial/peribronchiolar mononuclear infiltrates not observed in control animals but significance questionable)<br>(1) Mild peribronchiolar primarily mononuclear inflammatory infiltrates that occasionally extend into adjacent alveoar septa (1-3 cells thick) or alveolar spaces<br>(2) Mild to moderate, mononuclear to mixed inflammation (>3 cells thick) expands alveolar septa or spaces and/or occasionally obscures normal septal architecture<br>(3) Moderate mixed interstitial inflammation, and/or alveolar damage characterized by type I pneumocyte necrosis/loss with replacement by hemorrhage, fibrin, edema, and/or necrotic debris (reminiscent of hyaline membranes), and or scattered atypical/syncytial cells<br>(4) Marked alveolar inflammation (mixed), alveolar septal damage (as described above) and loss of normal septal architecture with frequent atypical/syncytial cells
**Type II pneumocyte hyperplasia (0-3)**	(0) None<br>(1) Scattered type II pneumocyte hyperplasia affecting less than 10% of the section<br>(2) Mild to moderate type II pneumocyte hyperplasia affecting 10-25% of the section (+/- atypical or multinucleated cells)<br>(3) Widespread type II pneumocyte hyperplasia and/or atypical/ multinucleated syncytial cells affecting greater than 25% of the section
**Total lung/alveolar score**	**0-11**
**Total regeneration score**	**0-6**
**Alveolar edema/protein/ hemorrhage/fibrin**	(0) absent; (1) present
**Vessels (% affected, 0-4)**	(0) None<br>(1) <10%<br>(2) 10-25%<br>(3) 25-50%<br>(4) >50%
	(0) None<br>(1) Multifocal perivascular edema and/or mild, primarily mononuclear perivascular inflammation (macrophages, lymphocytes +/- few scattered neutrophils)<br>(2) Moderate mononuclear to mixed perivascular inflammation, edema or fibrin, with leukocytes occasionally transmigrating the vessel wall, and/or multifocal endotheliitis<br>(3) Severe mixed perivascular inflammation frequently expanding/replacing [illegible]
